# Comprehensive analysis of tumor mutation burden and immune microenvironment in gastric cancer

**DOI:** 10.1042/BSR20203336

**Published:** 2021-02-26

**Authors:** Jie Yu, QianYun Zhang, MengChuan Wang, SiJia Liang, HongYun Huang, Lang Xie, ChunHui Cui, JinLong Yu

**Affiliations:** 1 Department of General Surgery, Zhujiang Hospital, Southern Medical University, Guangdong, Guangzhou, 510282, P.R. China; 2 Department of Medical Ultrasound, Guangzhou Women and Children’s Medical Center, Guangdong, Guangzhou 510623, P.R. China; 3 Department of Pharmacology, Cardiac and Cerebral Vascular Research Center, Zhongshan School of Medicine, Sun Yat-Sen University, Guangzhou 510080, P.R. China

**Keywords:** Gastric cancer, Immune cells infiltration, Prognosis, Tumor mutation burden

## Abstract

Tumor mutation burden (TMB) was a promising marker for immunotherapy. We aimed to investigate the prognostic role of TMB and its relationship with immune cells infiltration in gastric cancer (GC). We analyzed the mutation landscape of all GC cases and TMB of each GC patient was calculated and patients were divided into TMB-high and TMB-low group. Differentially expressed genes (DEGs) between the two groups were identified and pathway analysis was performed. The immune cells infiltration in each GC patient was evaluated and Kaplan–Meier analysis was performed to investigate the prognostic role of immune cells infiltration. At last, hub immune genes were identified and a TMB prognostic risk score (TMBPRS) was constructed to predict the survival outcome of GC patients. The relationships between mutants of hub immune genes and immune infiltration level in GC was investigated. We found higher TMB was correlated with better survival outcome and female patients, patients with T1-2 and N0 had higher TMB score. Altogether 816 DEGs were harvested and pathway analysis demonstrated that patients in TMB-high group were associated with neuroactive ligand–receptor interaction, cAMP signaling pathway, calcium signaling pathway. The infiltration of activated CD4^+^ memory T cells, follicular helper T cells, resting NK cells, M0 and M1 macrophages and neutrophils in TMB-high group were higher compared than that in TMB-low group and high macrophage infiltration was correlated with inferior survival outcome of GC patients. Lastly, the TMBPRS was constructed and GC patients with high TMBPRS had poor prognosis.

## Background

According to the statistics in 2018, over 1 million newly diagnosed cases and almost 800000 cancer-related deaths have made gastric cancer (GC) one of the most intractable diseases worldwide. Overall, GC is ranked third in terms of incidence and fifth in terms of mortality [1]. The only curative measure for GC patients is surgery [2]. However, most of the cases are diagnosed in advanced stage making complete resection impossible [3]. The prognosis of GC patients is also partially decided by whether lymph nodes were involved [4]. Chemotherapy before or after surgery was proved to increase the benefit of patients. Besides, monoclonal drugs target human epidermal growth factor receptor 2 (HER2) and vascular endothelial growth factor receptor 2 (VEGFR2) have also been applied in the clinical practice [5].

Recently, immunotherapy emerged as a rising star in the cancer treatment. The measures consist mainly of immune checkpoint inhibitors (ICIs), cancer vaccines, adoptive T-cell transfer therapy and cytokine therapy [6,7]. Major breakthrough was accomplished by immunotherapy so far. A phase 2 trial revealed that nivolumab (anti-PD-1 monoclonal antibody (mAb)) plus ipilimumab (anti-CTLA-4 mAb) could benefit the patients of malignant pleural mesothelioma [8]. The combination of nivolumab and ipilimumab therapy showed promising result in metastatic melanoma patients, especially in patients with negative expression of PD-L1 [9]. Chimeric antigen receptor (CAR) T cells therapy significantly changed the landscape of lymphoma therapy, improving the remission rate of lymphoma patients [10,11]. In addition, CAR-T therapy also offered potential benefit to pancreatic cancer patients [12]. A randomized clinical trial demonstrated that cancer vaccine in combination with docetaxel could remarkably enhance the progression-free survival of metastatic breast cancer patients [13].

It is worth noting that immunotherapy has also been playing a more and more important role in GC treatment. ATTRACTION-2 study revealed that patients with unresectable or recurrent GC treated with anti-PD-1 mAbs showed an objective response rate (ORR) of 11.2% [14]. Due to the extraordinary result brought by anti-PD-1 mAbs, this measure was incorporated into the third-line treatment for advanced GC in the Japanese guideline. CheckMate-032 study demonstrated that Ipilimumab (anti-CTLA-4 mAbs) plus nivolumab group (anti-PD-1 mAbs) showed a higher ORR than nivolumab alone group [15]. Although immunotherapy is a promising solution for GC patients, the response rate is still limited and novel biomarkers are urgently needed to identify the suitable subgroup of patients.

Tumor mutation burden (TMB) is defined as the non-synonymous somatic mutation number per megabase in cancer cells [16,17]. Several retrospective and prospective studies demonstrated that TMB could be a promising predictive biomarker for immunotherapy especially for ICIs efficacy. Researchers found that high frequency of non-synonymous mutation was associated with higher response rate in both melanoma and non-small cell lung cancer patients treated with ICIs [18,19]. A pan-cancer analysis showed that TMB was indeed correlated with ICIs treatment response rate [20]. Ten different cancers treated with ICIs were incorporated into the KEYNOTE-158 study and the study result revealed that high TMB was associated with improved ORR and progression-free survival [16]. A phase 2 trial (NCT02915432) also demonstrated that GC patients with high TMB gained significantly longer survival advantage than those with low TMB [21]. Therefore, it is worth understanding the TMB status of GC and its relevance with immune cells infiltration.

With the development of bioinformatics, many resources on TMB and immune microenvironment status were available on multiple databases such as The Cancer Genome Atlas (TCGA) and Gene Expression Omnibus (GEO) database. However, few researches investigate the relationship between them. Therefore, the present study was performed to evaluate the prognostic value of TMB and its association with immune cells infiltration in GC patients.

## Methods

### Transcriptome and somatic mutation data acquisition

We obtained transcriptome and somatic mutation data from Genomic Data Commons Data Portal of TCGA database (https://portal.gdc.cancer.gov/). Transcriptome profiles of all GC samples and relative adjacent gastric mucosa samples were downloaded in HTSeq-FPKM format. Somatic mutation data were downloaded in ‘Masked Somatic Mutation’ and processed by VarScan software. The ‘Maftools’ R package [22] was applied to visualize the mutation genes and classification and type of the mutation. The clinical characteristics of GC patients, which including age, gender, AJCC-TNM stage, pathologic stage, tumor grade and living status, were also downloaded from TCGA database.

### TMB scores calculation and prognostic analysis

TMB was defined as the total count of somatic mutation of genes which including base substitutions, insertions and deletions. In this research, TMB scores were defined as total number of somatic mutation variants/length of exons. We calculated the TMB scores (mutation frequency) by perl scripts based on JAVA8 platform. The TMB-high and TMB-low groups were defined by median TMB scores. The TMB scores and clinical characteristics of each GC patients were merged by R software. We used Kaplan–Meier analysis to measure the length of survival time and *P*-value was calculated through log-rank test. The correlation between TMB level and clinical characteristics was analyzed by Wilcoxon’s rank-sum test.

### Identification of differentially expressed genes and pathway enrichment analysis

GC patients were divided by TMB-high and TMB-low group according to the measures as we previously described. Differentially expressed genes (DEGs) were identified by ‘limma’ package and false discovery rate was set as 0.05. Heatmap of DEGs was created by ‘pheatmap’ package. The ‘org.Hs.eg.db’ package was utilized to annotate the DEGs. The Gene Ontology (GO) analysis and Kyoto Encyclopedia of Genes and Genomes (KEGG) analysis were performed by ‘clusterProfiler’, ‘enrichplot’, ‘ggplot2’ and both filters of *P*-value and q-value were set as 0.05. Gene Set Enrichment Analysis (GSEA) was performed by the software downloaded from its official website (https://www.gsea-msigdb.org/gsea) and ‘c2.cp.kegg.v6.2.symbols.gmt gene sets’ was selected as gene set database.

### CIBERSORT algorithm

CIBERSORT is an analytical algorithm developed to detect the abundances of cell types in a mixed cell population, using gene expression data [23]. Firstly, we prepared the data with ‘limma’ R package. Then we used CIBERSORT algorithm to analyze the immune cell composition in GC patients and visualization was performed by barplot. The violin plot was utilized to visualize the distribution of immune cell and Wilcoxon’s rank-sum test was used to evaluate the immune cells infiltration between different TMB groups.

### Identification of differentially expressed immune-related genes

We downloaded immune-related gene list from immport database (https://www.immport.org/shared/genelists) and altogether 2498 genes were obtained. Intersection of DEGs and immune genes was visualized by ‘VennDiagram’ package.

### Establishment of TMB prognostic risk score of differentially expressed immune-related genes

We merged differentially expressed immune-related genes with corresponding survival data and univariate Cox regression analysis was performed to find out the prognostic genes. Multivariate Cox regression analysis was performed to identify the independent risk gene. The TMB prognostic risk score (TMBPRS) was calculated with TMBPRS = Ʃ (χ_i_ × EXP_i_) and χ_i_ was the coefficient derived from the multivariate Cox regression analysis. GC patients were divided into high-risk group and low-risk group with threshold of median risk score. Kaplan–Meier analysis was performed to assess the survival status between the two groups. Receiver Operating Characteristic (ROC) curve was generated to evaluate the predictive value of TMBPRS.

### Timer database

The ‘SCNA’ module of Timer database (https://cistrome.shinyapps.io/timer/) was designed to compare different immune cells infiltration with different copy number variation (CNV) of a given gene. We used this module to detect the immune cells infiltration with different CNV of the TMB-related immune genes. Box plots were drawn to visualize the distribution of immune cell subset with different CNV and two-sided Wilcoxon’s rank-sum test was used to calculate the *P*-value between normal and each mutation group. We also utilized ‘Survival’ module to compare the survival status for immune infiltrates with Kaplan–Meier plots. *P*-value was calculated through log-rank test.

### Statistical analysis

The normalization of data and differential analysis was performed by ‘limma’ R package. Cox regression analysis and Kaplan–Meier analysis was performed by ‘survival’ R package. Wilcoxon’s rank-sum test is a nonparametric test and is used to detect the difference between two groups. All statistical analysis was carried out in R software (Version 3.6.3). *P*-value <0.05 was considered as statistically significant.

## Results

### Overview of the mutation status of GC patients

**Figure 1:**
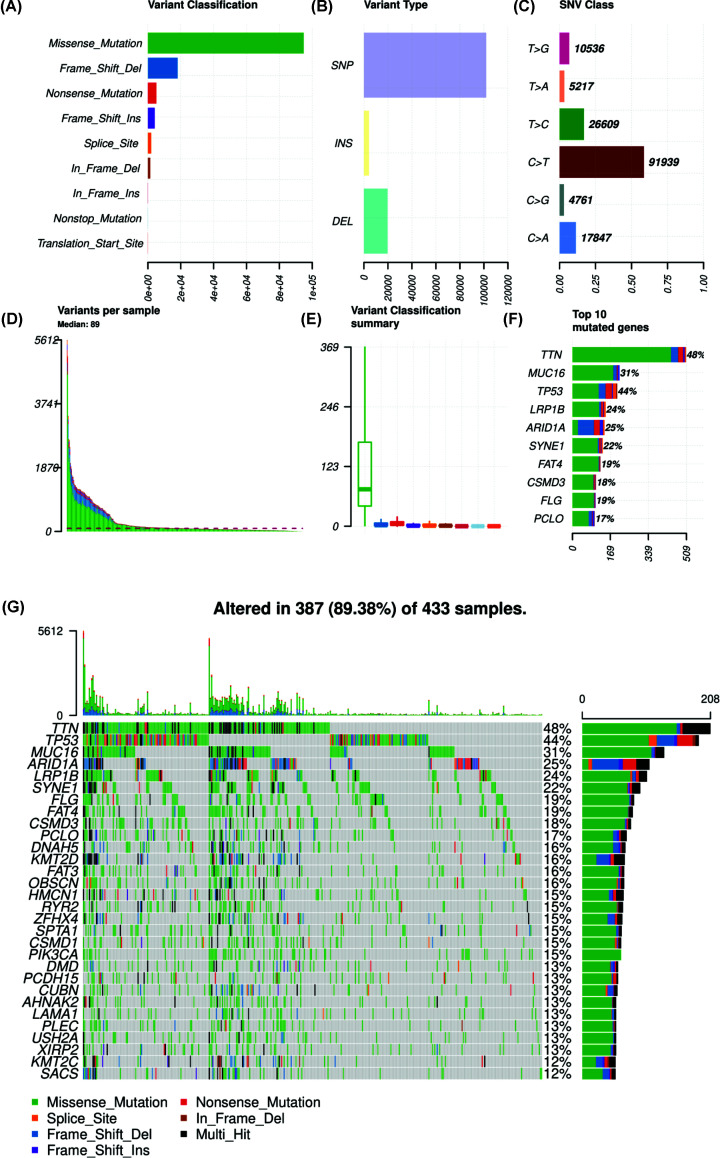
Summary of mutation landscape of GC samples from TCGA database (**A**–**C**) Mutation types based on different categories, where missense mutation was the most frequent component and SNP was the most common mutation type and C>T was the most common type of SNV. (**D**,**E**) TMB of each GC sample and its classification and the median variants number was 89. (**F**) Top ten mutated genes in GC samples and TTN, MUC16 and TP53 were the top three mutated genes. (**G**) Waterfall plot of mutation profiles of each gene in each sample. The legend at the bottom described the mutation types. The plot above the legends showed the mutation burden of each sample.

**Figure 2:**
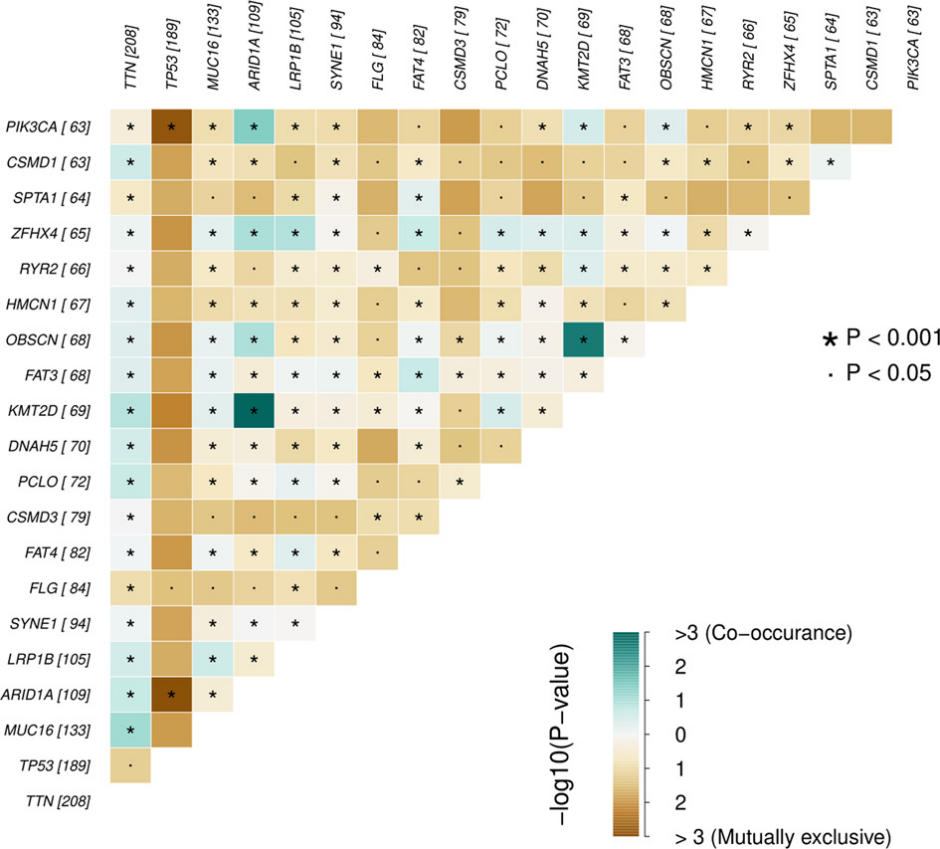
Pair of mutually exclusive or co-occurring mutated genes Pairwise Fisher’s Exact test was used to detect the statistical difference.

We obtained the somatic mutation data of GC patients from TCGA and chose the data processed by VarScan software. The ‘Maftools’ R package was utilized to visualize the landscape of mutation data of GC. According to variant classification, missense mutation, frameshift deletion and nonsense mutation were the first three mutations (Figure 1A). Single nucleotide polymorphism was the most common mutation type, followed by deletion and insertion (Figure 1B). Among the single nucleotide variants (SNVs) class, C>T was the most common mutation (Figure 1C). We also countered the number of mutations in each sample and the summary of mutation was visualized in box plot (Figure 1D,E). Top ten mutated genes in GC were also demonstrated in percentage form, including TTN (48%), MUC16 (31%), TP53 (44%), LRP1B (24%), ARID1A (25%), SYNE1 (22%), FAT4 (19%), CSMD3 (18%), FLG (19%) and PCLO (17%). Mutation of each gene in different samples is shown in waterfall plot (Figure 1G). Besides, genes mutated in more than 30 samples were shown by Genecloud plot (Supplementary Figure S1). The correlation of mutated genes is shown in Figure 2 and deep green squares indicate co-occurrence while brown squares indicate mutually exclusive.

### Assessment of TMB level and prognostic analysis

**Figure 3:**
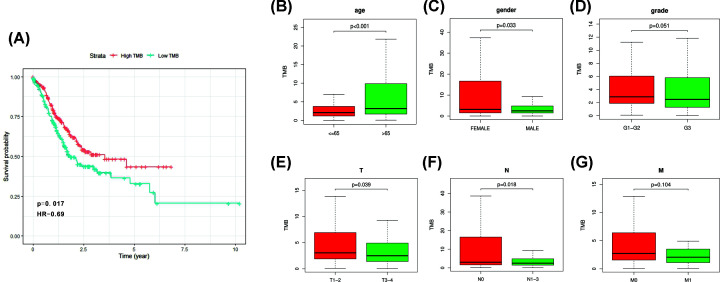
Prognostic value of TMB and its association with clinical characteristics (**A**) Higher TMB level was correlated with better survival outcome of GC patients, *P*=0.017. (**B**,**C**,**E**,**F**) Higher TMB was associated with lower age, female, lower AJCC-T stage and lower AJCC-N stage. (**D**,**G**) No statistical difference was observed between TMB and tumor grade and AJCC-M stage.

We calculated the TMB score of each GC sample by perl script. All GC samples were divided into TMB-high and TMB-low group according to median TMB score. Kaplan–Meier analysis was performed to evaluate the survival status between different groups. We found that high TMB score was correlated with better survival outcome (Figure 3A). We matched TMB status with clinicopathological characteristics (Table 1) of GC patients and found that GC patients with age > 65 years had higher TMB score. Besides, female patients and patients with T1-2 and N0 had higher TMB score than the others (Figure 3B,C,E,F). There was no difference between TMB score and tumor grade and AJCC-M stage (Figure 3D,G).

**Table 1:** Clinical characteristics of TCGA GC patients

Variables	TCGA cohort (*n*=443)
Status	
Alive	272 (61.4)
Dead	171 (38.6)
Age	66 ± 10.76
Gender	
Female	158 (35.6)
Male	285 (64.4)
AJCC‐T	
T1	23 (5.2)
T2	93 (21.0)
T3	198 (44.7)
T4	119 (26.9)
TX	10 (2.2)
AJCC‐N	
N0	132 (29.8)
N1	119 (26.9)
N2	85 (19.2)
N3	88 (19.9)
NX	17 (3.8)
Unknown	2 (0.4)
AJCC‐M	
M0	391 (88.3)
M1	30 (6.8)
MX	22 (4.9)
Pathologic stage	
I–II	192 (43.3)
III–IV	224 (50.6)
Unknown	27 (6.1)
Tumor grade	
G1–G2	171 (38.6)
G3	263 (59.4)
GX	9 (2.0)

### Comparison of DEGs between TMB-high and TMB-low group and pathway analysis

**Figure 4:**
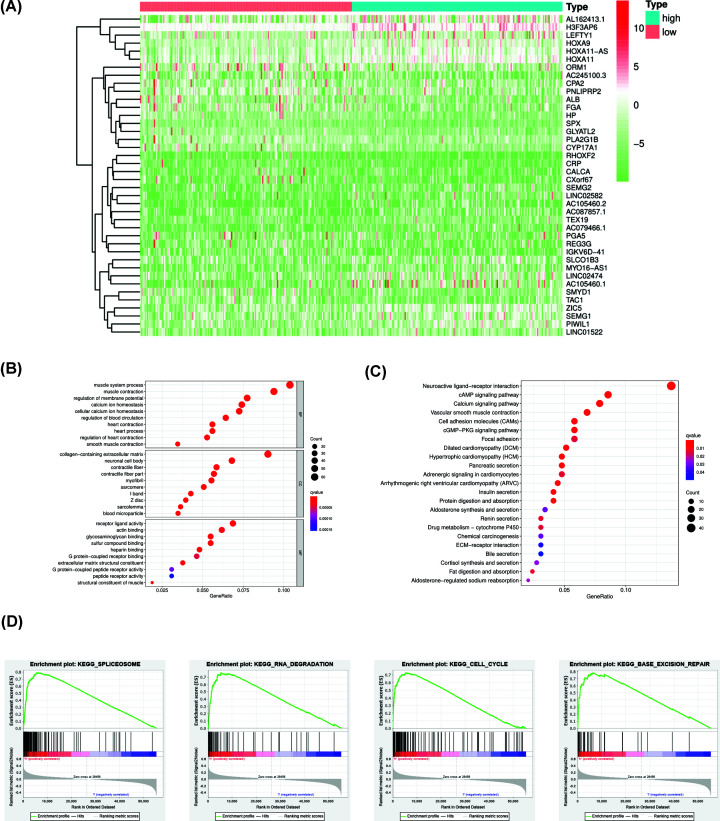
Comparison of DEGs between TMB-high and TMB-low groups and pathway analysis (**A**) Top 40 DEGs between TMB-high and TMB-low group were shown in heatmap. (**B**,**C**) GO and KEGG analyses of DEGs between TMB-high and TMB-low groups. (**D**) GSEA indicated that high TMB was correlated with spliceosome, RNA degradation, cell cycle and base excision repair.

As we previously described, GC patients were divided into two groups. We compared the DEGs by using ‘limma’ package with |Fold Change| >1 and 816 DEGs were harvested. DEGs between two groups were visualized in heat map (Figure 4A). GO and KEGG analysis were also performed and these DEGs in TMB-high group were mainly involved in neuroactive ligand–receptor interaction, cAMP signaling pathway, calcium signaling pathway (Figure 4B,C and Table 2). GSEA indicated that high TMB level was correlated with splicesome, RNA degradation, cell cycle and base excision repair (Figure 4D). In addition, low TMB level was associated with arachidonic acid metabolism, calcium signaling pathway, neuroactive ligand–receptor interaction and vascular smooth muscle contraction (Supplementary Figure S2).

**Table 2:** KEGG analysis of the DEGs in TMB-high group

Description	Bg ratio	*P*-value	*P*.adjust	q-value
Neuroactive ligand–receptor interaction	340/8040	2.58E-11	6.64E-09	0.00000001
Vascular smooth muscle contraction	132/8040	4.90E-08	6.30E-06	0.00000537
cAMP signaling pathway	216/8040	2.44E-07	2.09E-05	0.00001779
Calcium signaling pathway	193/8040	4.46E-07	2.86E-05	0.00002440
Dilated cardiomyopathy (DCM)	96/8040	1.62E-06	8.33E-05	0.00007096
Arrhythmogenic right ventricular cardiomyopathy (ARVC)	77/8040	3.33E-06	0.00014	0.00011966
Hypertrophic cardiomyopathy (HCM)	90/8040	3.83E-06	0.00014	0.00011966
Pancreatic secretion	102/8040	1.70E-05	0.000546	0.00046522
Cell adhesion molecules (CAMs)	148/8040	2.40E-05	0.000685	0.00058390
Insulin secretion	86/8040	5.79E-05	0.001487	0.00126717
cGMP-PKG signaling pathway	167/8040	0.000112	0.002621	0.00223331
Protein digestion and absorption	95/8040	0.000155	0.00331	0.00281991
Renin secretion	69/8040	0.000811	0.014904	0.01269720
Fat digestion and absorption	43/8040	0.000812	0.014904	0.01269720
Focal adhesion	201/8040	0.000996	0.016124	0.01373677
Adrenergic signaling in cardiomyocytes	149/8040	0.001004	0.016124	0.01373677
Drug metabolism - cytochrome P450	72/8040	0.001108	0.016744	0.01426520
Aldosterone-regulated sodium reabsorption	37/8040	0.001959	0.027971	0.02382945
Cortisol synthesis and secretion	65/8040	0.002295	0.031038	0.02644201
Aldosterone synthesis and secretion	98/8040	0.002842	0.036521	0.03111326
Chemical carcinogenesis	83/8040	0.003022	0.036981	0.03150577
ECM–receptor interaction	88/8040	0.004487	0.052421	0.04465959
Bile secretion	90/8040	0.005209	0.058203	0.04958528

### Immune cell infiltration in TMB-high and TMB-low GC patients

**Figure 5:**
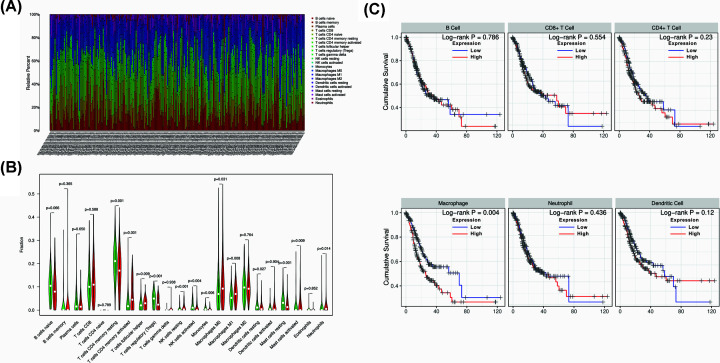
Immune cells infiltration between TMB-high and TMB-low groups and survival analysis of immune cells (**A**) Twenty-two types of immune cells infiltration status in each GC sample. (**B**) The comparison of immune cells infiltration between TMB-high and TMB-low groups. (**C**) Kaplan–Meier analysis of different immune cells and high macrophage infiltration was correlated with worse survival outcome of GC patients.

As we have separated two groups of GC patients according to TMB level, we wanted to investigate the immune cells infiltration between the two groups. By using ‘CIBERSORT’ R package, we compared 22 immune cells in TMB-high and TMB-low groups. The fraction of 22 immune cells in each GC patients were shown in Figure 5A and different colors represented different immune cell type. Furthermore, the violin plot was utilized to visualize the immune cell proportion. Wilcoxon’s rank-sum test revealed that the infiltration of activated CD4^+^ memory T cells, follicular helper T cells, resting NK cells, M0 and M1 macrophages and neutrophils in TMB-high group were higher compared than that in TMB-low group (Figure 5B). The absolute abundance of each immune cell type in each patient was shown in Supplement Table S1. In order to further investigate the prognostic role of immune cells, we constructed a Cox regression model in GC samples and the formula was demonstrated as follow: Surv (STAD) ∼ B cell + CD8^+^ T cell + CD4^+^ T cell + Macrophage + Neutrophil + Dendritic. The result showed that macrophage infiltration was the only risk factor for GC patients (HR = 293.055, *P*<0.001 Table 3). Kaplan–Meier analysis was also performed and the result showed that high macrophage was correlated with inferior survival outcome of GC patients (Figure 5C).

**Table 3:** Multivariate Cox regression analysis of immune infiltration cells in GC

Cell types	Coef	HR	95%CI_l	95%CI_u	*P-*value	sig
B_cell	3.262	26.096	0.419	1625.426	0.122	-
CD8_T cell	−2.040	0.130	0.009	1.966	0.141	-
CD4_T cell	−3.825	0.022	0.000	1.763	0.088	-
Marcophage	5.680	293.055	15.915	5396.255	0.000	*
Neutrophil	−0.629	0.533	0.003	88.445	0.809	-
Dendritic	1.506	4.510	0.401	50.764	0.223	-

*P<0.05.

### Identification of immune-related DEGs and TMBPRS establishment

**Figure 6:**
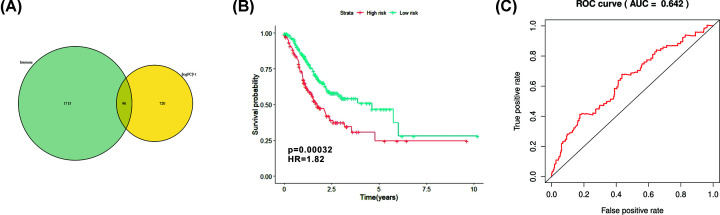
Identification of hub immune genes and construction of TMBPRS (**A**) Identification of differentially expressed immune-related genes through Venn plot. (**B**) Assessment of TMBPRS in GC patients and patients with high TMBPRS had worse survival outcome (*P*=0.00032). (**C**) ROC plot of TMBPRS with AUC = 0.642.

We downloaded immune-related genes from immport database and ‘VennDiagram’ package was utilized to screen out 96 immune-related genes (Figure 6A). Univariate Cox regression analysis was performed and further identified 12 prognostic genes (Table 4). TMBPRS was constructed basing on multivariate Cox regression analysis and model was demonstrated as follow: PRS = (0.001763 × APOD + 0.033231 × FGF7 + 0.107249 × AMHR2 + 0.067987 × NPR3) (Table 5). And then we calculated the TMBPRS of each GC patient and patients were divided into high-risk and low-risk groups with the cutoff value of median. Kaplan–Meier analysis was performed and the result showed that GC patients with high risk had worse survival outcome (Figure 6B). The ROC curve of 1-year overall survival (OS) prediction was drawn with area under curve (AUC) = 0.642 (Figure 6C).

**Table 4:** Univariate Cox regression analysis of immune-related DEGs

	HR	HR.95L	HR.95H	coxPvalue
SLC22A17	1.067292	1.023803	1.112629	0.002153
APOD	1.002423	1.001157	1.003692	0.000175
CMA1	1.155618	1.043777	1.279444	0.005354
FGF7	1.048351	1.016115	1.081609	0.003044
OGN	1.006824	1.001391	1.012288	0.013767
AMHR2	1.109999	1.038009	1.186982	0.002286
GHR	1.215142	1.071306	1.37829	0.002433
GLP2R	1.412913	1.092291	1.827648	0.008483
NPR3	1.073467	1.009318	1.141694	0.024136
PTGER3	1.149958	1.018448	1.298451	0.024135
PTGFR	1.198121	1.043102	1.376179	0.010561
PTH1R	1.440015	1.056238	1.963235	0.021113

**Table 5:** Multivariate Cox regression analysis of immune-related DEGs

Id	Coef	HR	HR.95L	HR.95H	Cox *P*-value
APOD	0.001763	1.001765	1.000301	1.00323	0.018081
FGF7	0.033231	1.03379	0.998769	1.070039	0.058773
AMHR2	0.107249	1.113211	1.037874	1.194017	0.002702
NPR3	0.067987	1.070352	0.998354	1.147541	0.055671

### Association between CNV of TMB-related immune genes and immune cell infiltrate

**Figure 7:**
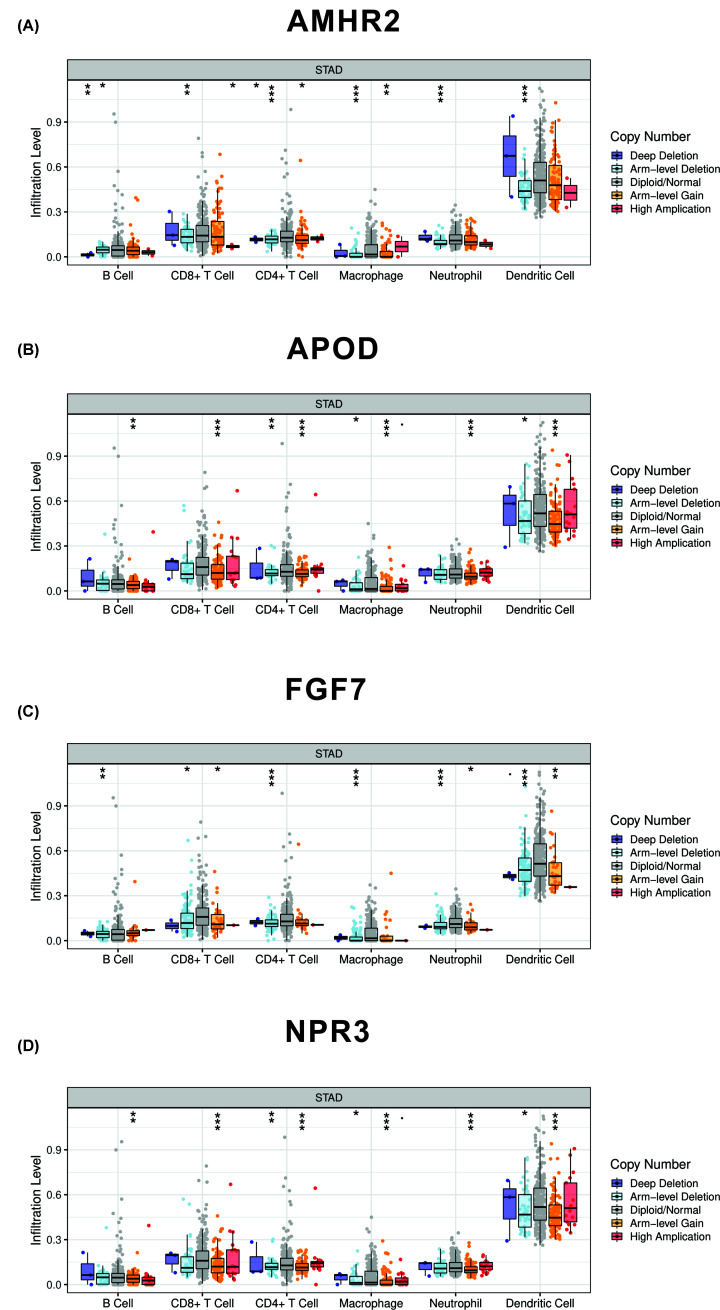
Correlation between mutants of four hub immune genes and immune cells infiltration (**A**) Correlation between mutants of AMHR2 and immune cells infiltration. Arm-level deletion of AMHR2 was correlated with reduced infiltration level of B cell, CD8^+^ T cell, CD4^+^ T cell, macrophage, neutrophil and dendritic cell. (**B**) Correlation between mutants of APOD and immune cells infiltration. Arm-level gain of APOD was correlated with reduced infiltration level of B cell, CD8^+^ T cell, CD4^+^ T cell, macrophage, neutrophil and dendritic cell. (**C**) Correlation between mutants of FGF7 and immune cells infiltration. Arm-level deletion of FGF7 was correlated with reduced infiltration level of B cell, CD8^+^ T cell, CD4+ T cell, macrophage, neutrophil and dendritic cell. (**D**) Correlation between mutants of NPR3 and immune cells infiltration. Arm-level gain of NPR3 was correlated with reduced infiltration level of B cell, CD8^+^ T cell, CD4^+^ T cell, macrophage, neutrophil and dendritic cell.

As we previously described, we used ‘VennDiagram’ package to identify the intersection between DEGs and immune-related genes and 96 differentially expressed immune-related genes were harvested. Further univariate analysis was applied to identify genes associated with prognosis. At last, we identified four hub immune genes (*APOD, FGF7, AMHR2, NPR3*) that were correlated with TMB. We then further investigated the association between mutants of these hub immune genes and immune cell infiltrate. The ‘SCNA’ module of Timer database was used to analyze the association and B cell, CD8^+^ T cell, CD4^+^ T cell, macrophage, neutrophil and dendritic cell were incorporated into the analysis (Figure 7). Besides, all four hub immune genes were also analyzed by Kaplan–Meier method in TCGA and K–M plotter database (Supplementary Figure S3). The methylation status of the four hub immune genes were also assessed in Supplementary Figure S4.

## Discussion

Immunotherapy has brought a revolutionary advance in the field of oncology and ICIs played a pivotal role in it. The mAb which targets PD-1 and CTLA-4 are the most extraordinary examples of cancer immunotherapy. Human PD-1 is expressed on the surface of T cells and binds to the PD-L1/PD-L2 that are present on antigen-presenting cells (APCs). The PD-1/PD-L1 axis was found to negatively regulate T-cell activation and its immunosuppression effect is mainly through inhibitory signaling pathway in effect T cells and regulatory T (T_reg_) cells [24]. Since pembrolizumab and nivolumab were approved by FDA in 2014, they have changed the way of cancer therapy. A phase 3 clinical trial demonstrated that pembrolizumab plus chemotherapy drugs had prolonged the OS of non-small-cell lung cancer patients compared with chemotherapy alone [25]. Recent study focuses on melanoma also found that patients treated with nivolumab plus ipilimumab remarkably improved their OS with 52% of them survived more than 5 years [26]. Another clinical trial showed that atezolizumab plus nab-paclitaxel improved the progression-free survival of metastatic triple-negative breast cancer patients [27]. Besides, myriad of clinical trials demonstrated the efficacy of anti-PD1/PD-L1 in many cancer types such as urothelial carcinoma, renal cell carcinoma, small-cell lung cancer [28–30].

Although spectacular result made with ICIs, complicated microenvironment of different organs makes it difficult to predict which patient will benefit. Several markers such as PD-1/PD-L1 expression [16], microsatellite instability [31] and CD8^+^ T cell infiltration [32] have been developed to recognize appropriate patients but their effects were limited. Therefore, finding better marker to optimize the therapeutic effects of ICIs is of vital importance.

TMB, a promising marker for ICIs treatment, has been found to play a vital role in predicting the response of immunotherapy. Ready et al. found that non-small cell lung cancer patients with high TMB had better response rate and prolonged progression-free survival when treated with nivolumab plus low-dose ipilimumab despite PD-L1 expression [33]. Among 22 colorectal patients treated with PD-1/PD-L1 inhibitors, all TMB-high patients responded while 6 out of 9 TMB-low patients progressed [34]. Besides, TMB also been demonstrated its effect in various of cancer such as breast cancer, melanoma, urothelial carcinoma and so on [19,35,36]. Our study showed that GC patients with higher TMB had better survival outcome and this finding was in accordance with other cancer research. In addition, we outlined the TMB-related characteristics of GC patients and observed that TMB-high was correlated with younger age, female, T1-T2 and N0 in GC cases.

In the current study, we calculated the TMB score of each GC patients by perl. GC patients then were divided into TMB-high and TMB-low groups. By comparing the DEGs between TMB-high and TMB-low groups, we identified 816 DEGs. GO and KEGG pathway analyses indicated that these DEGs were mainly involved in neuroactive ligand–receptor interaction, cAMP signaling pathway, calcium signaling pathway and so on. Further uni- and multivariate Cox analyses indicated that AMHR2, APOD, FGF7 and NPR3 were the hub immune genes and correlated with the prognosis of GC patients. We also found that mutant of these genes was correlated with the immune infiltrates. Immune cells infiltration such as B cell, CD8^+^ T cell, CD4^+^ T cell, macrophage, neutrophil and dendritic cell were inhibited by the mutation of these genes. To be specific, Arm-level deletion of AMHR2 and FGF7 were associated with reduced infiltration of immune cells. However, Arm-level gain of APOD and NPR3 were associated with reduced infiltration of immune cells.

APOD is an encoding gene which encodes a component of high-density lipoprotein. Researchers found that high expression of APOD was correlated with worse survival outcome of breast cancer patients [37]. Another group also reported that APOD was highly expressed in prostate cancer and high grade prostatic intraepithelial neoplasia compared with adjacent normal tissue [38]. FGF7 belongs to fibroblast growth factor (FGF) family and possess mitogenic and cell survival activities. Zhu et al. reported that FGF7 could promote breast cancer progression through AKT signaling pathway [39]. In GC, several studies indicated that FGF7 might play a role in GC cell proliferation and metastasis [40,41]. The product of gene *NPR3* encodes one of the natriuretic peptide receptors and responsible of clearing natriuretic peptides. Previous study demonstrated that high expression of NPR3 was correlated with poor prognosis of colorectal patients [42].

It is widely recognized that immune cells infiltration status had prognostic value in multiple cancer. We compared 22 immune cells between TMB-high and TMB-low group and found that CD4^+^ memory T cells, follicular helper T cells, resting NK cells, M0 and M1 macrophages and neutrophils were differently infiltrated in the two groups. In order to further investigate whether this difference in the two groups would affect the survival outcome of GC patients, we performed the Cox regression analysis. The result demonstrated that high macrophage infiltration was associated with worse survival outcome of GC patients. Similar conclusion was drawn by Su et al. that high density of macrophage predicted a poor survival outcome of GC patients [43]. Several studies investigated the interaction of macrophage and GC cell and found that macrophage might play a role in promoting GC cell proliferation, metastasis, angiogenesis, chemoresistance and immune invasion [44–47].

Finally, a prognostic algorithm (TMBPRS) was constructed according to the Cox regression analysis and patients with high TMBPRS had worse survival outcomes. However, the AUC of this algorithm was only 0.642 and therefore large data research was needed to improve the predictive effect.

However, there were some limitations in the present study: (a) lack of basic experiment such as immunohistochemistry to identify the correlation between four hub immune genes and immune cells infiltration and (b) large clinical samples are needed to validate the prognostic effect of TMBPRS.

## Conclusions

Higher TMB was correlated with better survival outcome of GC patients. High macrophage infiltration predicted worse prognosis of GC patients.

## Supplementary Material

Supplementary Figures S1-S4

## Data Availability

The authors confirm that the data supporting the findings of the present study are available within the article [and/or] its supplementary materials.

## Abbreviations

AUC: area under curve
CAR: chimeric antigen receptor
CNV: copy number variation
CTLA-4: cytotoxic T-lymphocyte-associated protein 4
DEG: differentially expressed gene
FGF: fibroblast growth factor
GC: gastric cancer
GO: Gene Ontology
GSEA: Gene Set Enrichment Analysis
ICI: immune checkpoint inhibitor
KEGG: Kyoto Encyclopedia of Genes and Genomes
mAb: monoclonal antibody
NK: natural killer
ORR: objective response rate
OS: overall survival
PD-1: programmed death-1
PD-L1: programmed death ligand 1
ROC: receiver operating characteristic
TCGA: The Cancer Genome Atlas
TMB: tumor mutation burden
TMBPRS: TMB prognostic risk score
TNM: Tumour, Node, Metastasis
