# Supplementary figures and images for: Comprehensive analysis of tumor mutation burden and immune microenvironment in gastric cancer
Source: Biosci Rep. 2021 Feb 26;41(2):BSR20203336. doi: 10.1042/BSR20203336 (PMC7921293; doi:10.1042/BSR20203336)

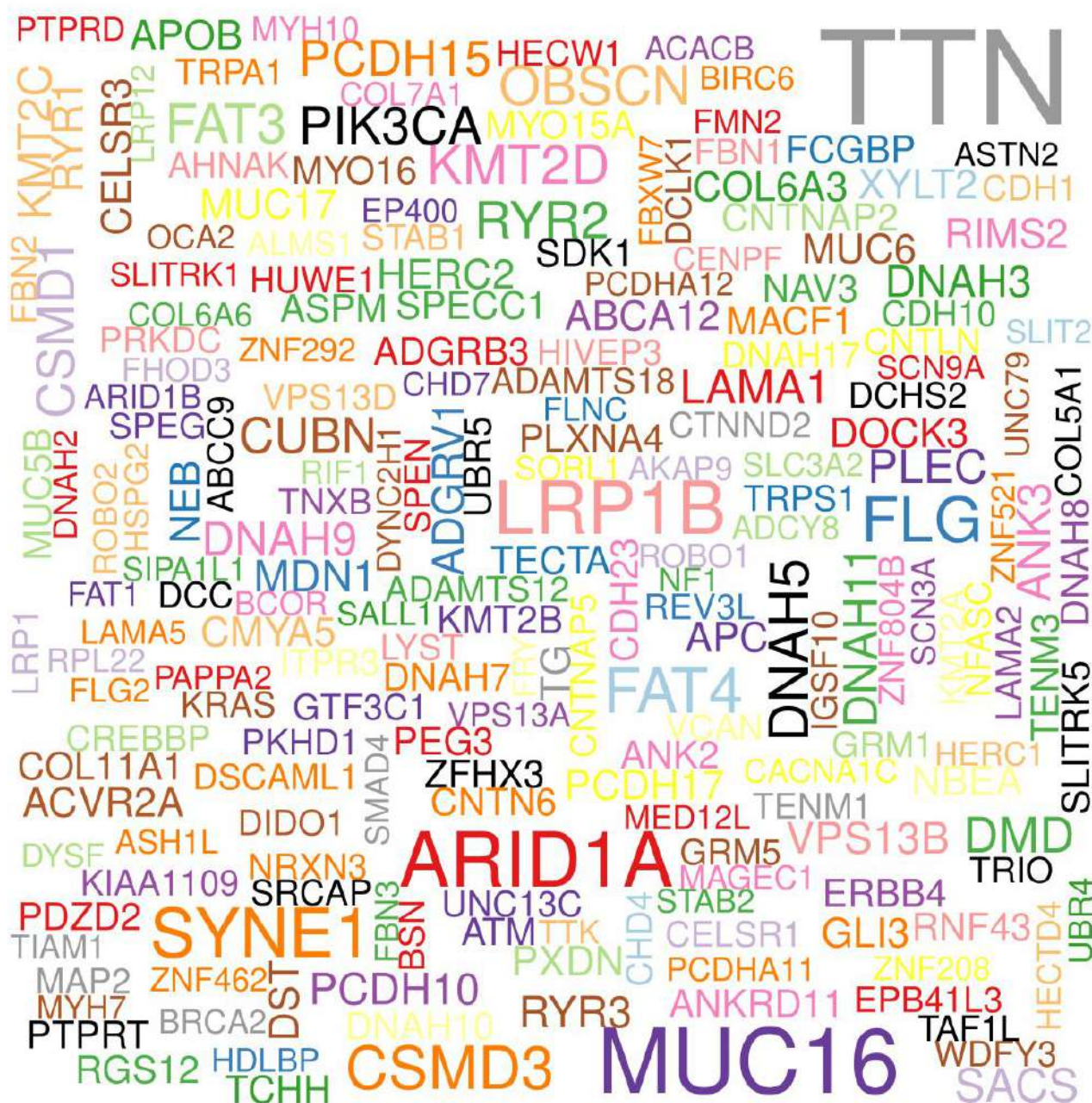

A

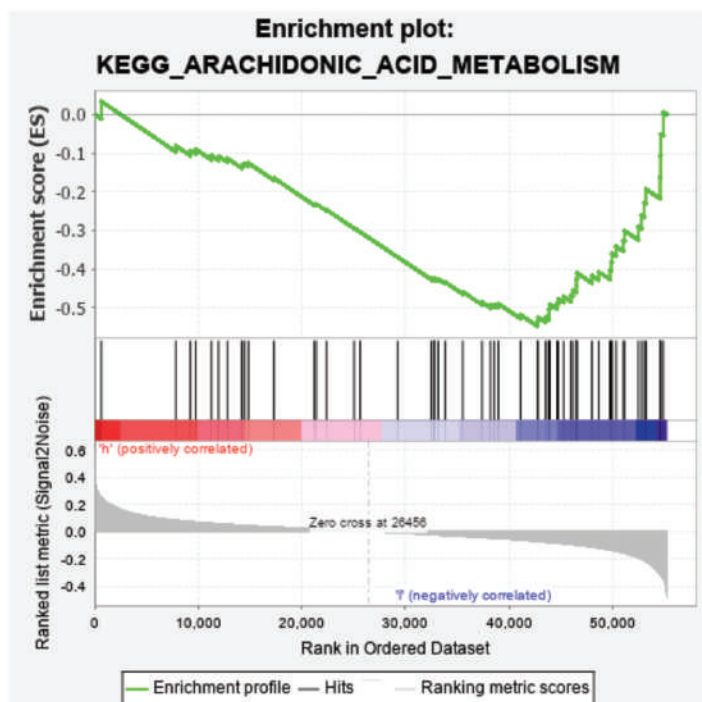

B

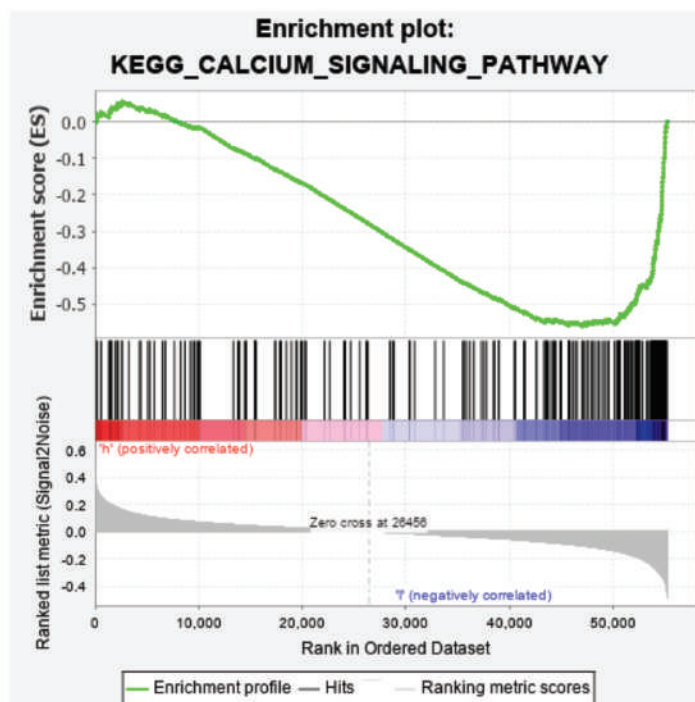

C

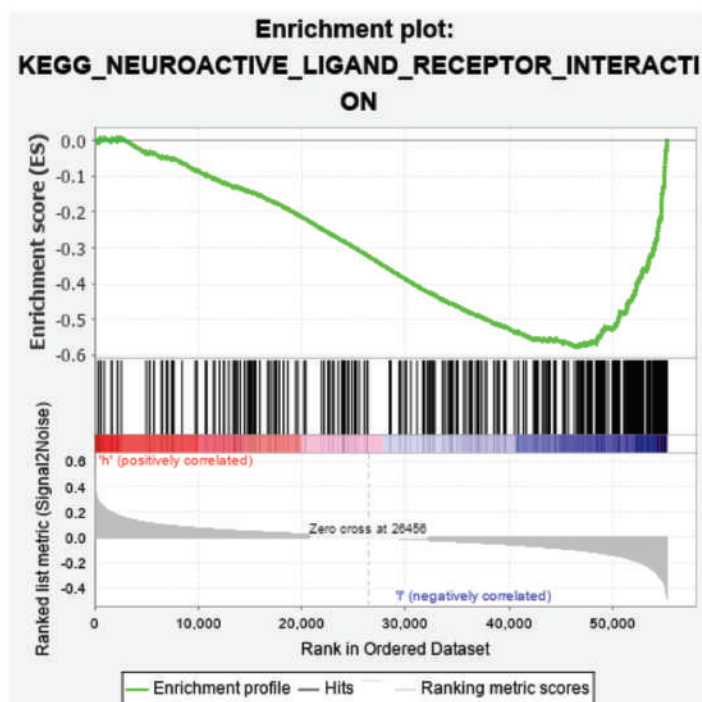

D

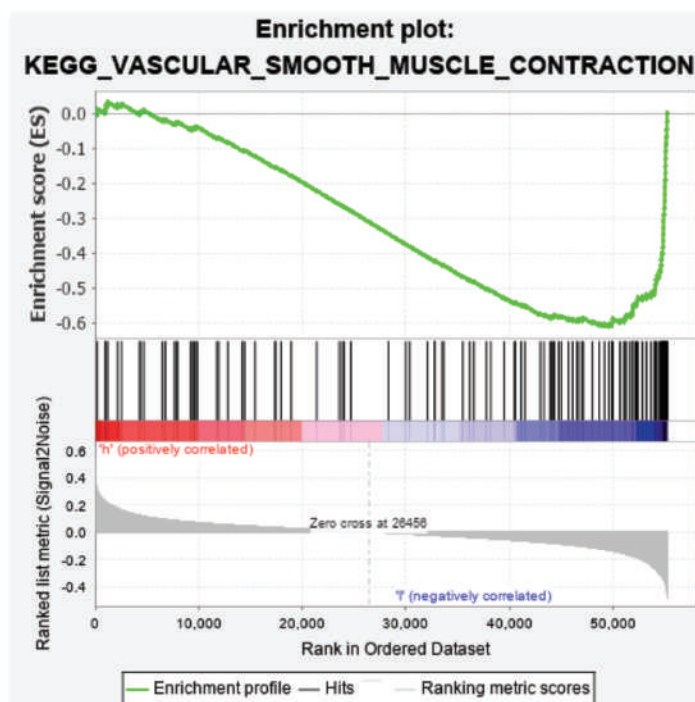

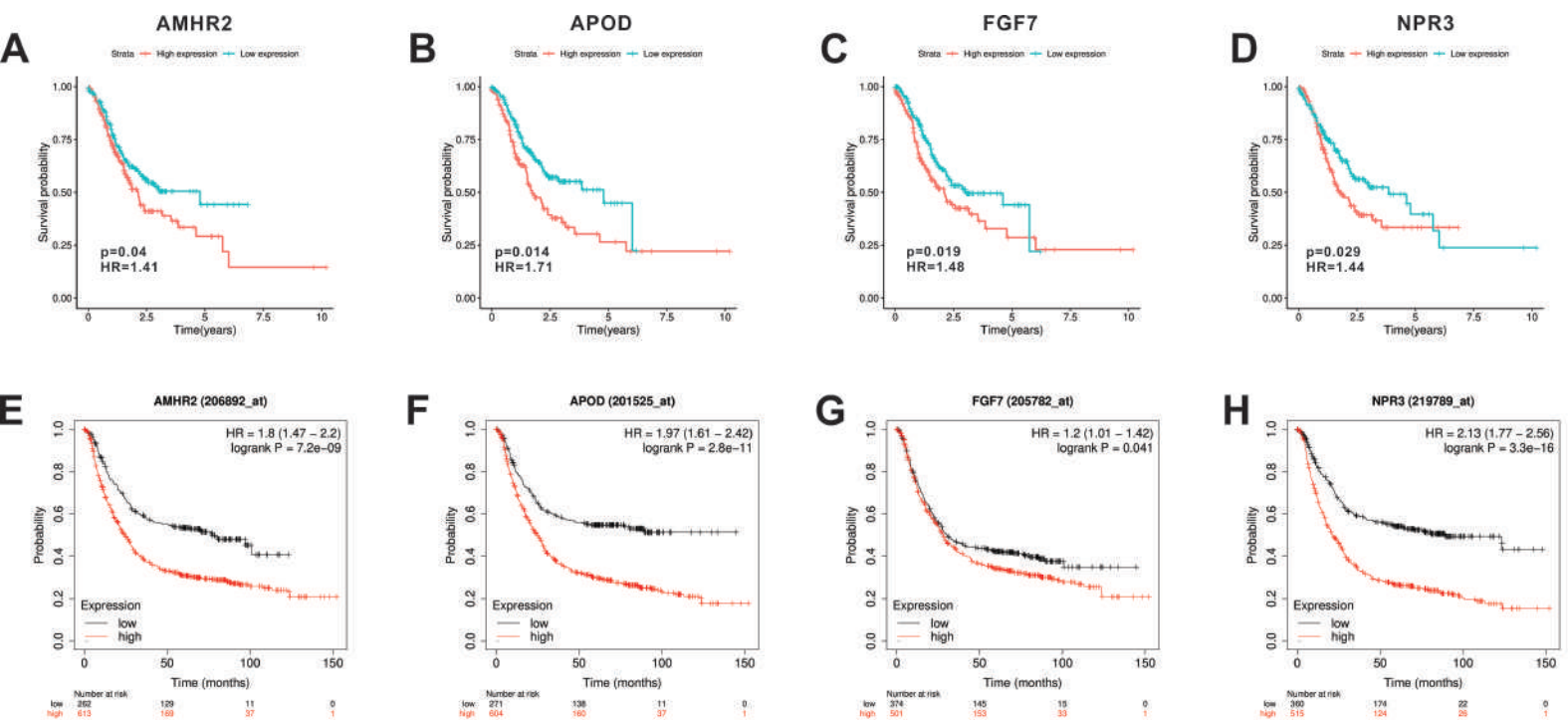

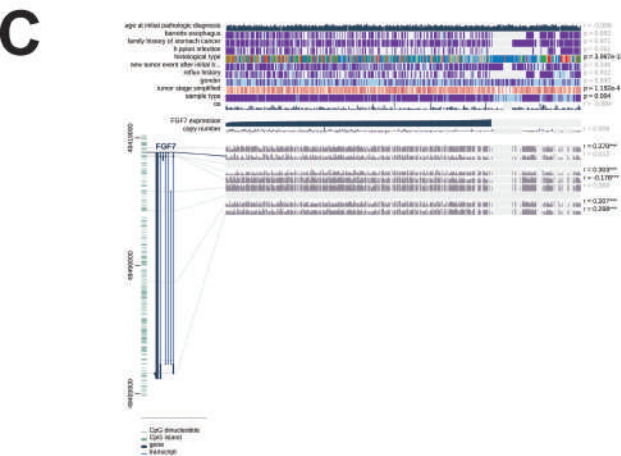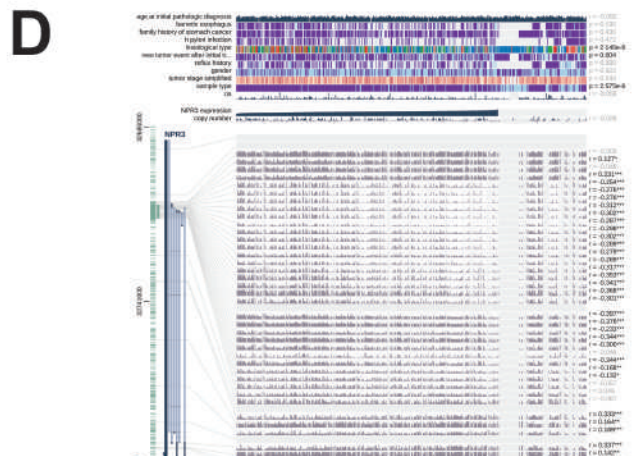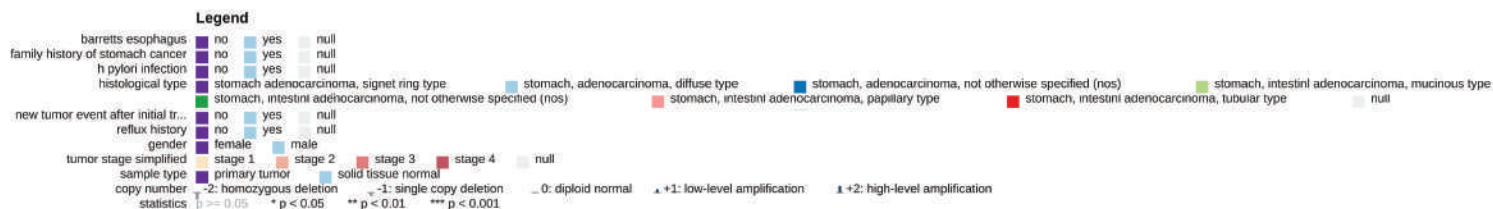

Supplement: Supplementary Figures S1-S4 [file BSR-2020-3336_supp.pdf]
